# Physiology in Education

**Published:** 1899-04-29

**Authors:** 


### The Hospital

A Journal of

The Medical Sciences and Hospital Administration.

Vol. XXVI.—No. 657.] Edited by Sir Henry Burdett, K.C.B., and Solomon C. Smith, M.D., M.R.C.P. [April 29, 1899.

### PHYSIOLOGY IN EDUCATION.

Among the many problems which await solution at the hands of the physiologists of the future, there is none more curious, more interesting, or more calculated to modify the destinies of the human race, than the determination of the degree in which the moral and intellectual development of the individual, and hence of the aggregate of individuals constituting a community, admit of being altered and controlled by environment, and especially by that adjustment of environment to the training of the young which is commonly described as education. That "blessed word," quite irrespectively of any precise knowledge of its meaning, or of the limitations placed by Nature around the influence of the processes which it is used in common parlance to denote, affects many politicians and public men as powerfully as "Mesopotamia" did the devout old lady of the familiar story of our youth; and, more especially when it follows such an adjective as "secondary," or "higher," or "technical," appears to represent to them the one agency by which England is to retain, or even to improve, her place in the scale of nations. Nevertheless, if we carefully analyse even the most superficial aspects of the infinite diversities of character, of temperament, and of ability, it becomes very difficult, to say the least, to ascertain with any approach to certainty the conditions, either inherited or accidental, to which they may with any approach to probability be ascribed. The merit of having contributed to the formation of a great man is claimed with equal confidence by the family of his mother, by the family of his father, by the schools or universities at which he was taught, and on behalf of the circumstances which first afforded an opportunity for the display of his powers. Assuming all these elements to have been contributory to the general result, there is still nothing to show their respective shares in its production, or the manner and degree in which it might have been modified by any change in one or more of them. "When there is nothing great to be done, there can be no great men;" but it is problematical how far the assigned condition can ever be fulfilled; and it is at least certain that, as a rule, Nature is sufficiently prodigal of power to supply it whenever an opportunity arises for its display. It may fairly be assumed that, if not the "mute inglorious Milton," yet that the "village Hampden" and the "guiltless Cromwell" are always with us, and are regarded by their respective neighbours as commonplace people, respectably following ordinary occupations. Nothing manifest or acknowledged or purposive has ever been done to differentiate them from their fellows, or to prepare them for an emergency which may never arise. The routine of military service furnishes a Pollock or a Havelock; the routine of naval service furnishes a Nelson. The great masters of science or of learning spring up from among numbers of their fellows who enjoy the same, or, it may be, even greater advantages or opportunities; and no one can explain in what respects, other than in achievement, they differ from the undistinguished. More than one attempt has been made by theorists to produce desired results by special methods of education, but always without success. Perhaps the most careful was that of Day, the author of "Sandford and Merton," who took two young girls from an orphanage in order to train them, and with a view to marry the one who most nearly realised his anticipations; but whose "Lucretia," being "invincibly stupid," was apprenticed to a milliner, and ultimately married a linendraper; while "Sabrina," who started when hot sealing-wax was dropped on her arm, screamed when a pistol loaded with blank cartridge was fired at her, betrayed secrets meant to test her reticence, and cared little for books of science, was permitted by her baffled preceptor to become the wife of a barrister. There are facts which, taken alone, would support the inference that the influence of inheritance is all powerful; there are others which appear to show that it is only trivial. It may perhaps be urged in favour of extended schooling that it will increase the number of persons who, in each generation, are qualified to avail themselves of opportunities; but, at least upon any basis of assured knowledge, it would be impossible to claim for it a more potent or more extended operation. We were once told by a large shipper of watches that, if he gave an order to a manufacturer for fifty of the best that could be made, he would be likely to receive forty-eight that would pass muster by all ordinary tests, one that was super-excellent, and one that was hopelessly defective. He explained this as a result of the want of absolute accuracy of measurement. May it not be said that a school, in like manner, turns out an occasional genius, an occasional failure, and a fair average of mediocrity? The problem which awaits physiology is to tell us by what means the failure may be elevated towards mediocrity, or the mediocrity towards genius. The labours of Golgi and his followers, and the recently-acquired knowledge of the structure of the neuron, may not improbably furnish the foundation of some real insight into the physical basis of mental operations, and thus, ultimately, into the methods by which they may be guided or controlled. At present the minimum of information upon these subjects is probably represented by that which is in the possession of the average schoolmaster, who, as a rule, does not entertain even a suspicion of his own abysmal ignorance of the conditions which determine the results of his proceedings.

